# Therapeutic Efficacies of* Artemisia capillaris* Extract Cream Formulation in Imiquimod-Induced Psoriasis Models

**DOI:** 10.1155/2018/3610494

**Published:** 2018-08-19

**Authors:** Song Yi Lee, Suyeong Nam, Sungyun Kim, Ja Seong Koo, In Kee Hong, Hill Kim, Sangin Han, Minji Kang, Heejung Yang, Hyun-Jong Cho

**Affiliations:** ^1^College of Pharmacy, Kangwon National University, Chuncheon, Gangwon 24341, Republic of Korea; ^2^R&D Center, Radiant Ltd., Chuncheon, Gangwon 24398, Republic of Korea; ^3^Hankook Korus Pharm. Co. Ltd., Chuncheon, Gangwon 24398, Republic of Korea

## Abstract

A cream formulation containing* Artemisia capillaris* (AC) extract (ACE) was developed for psoriasis therapy. Although ACE can be dissolved in organic solvents, its topical application is restricted because of toxicities. Therefore, a cream formulation was developed for the convenient and safe local application of ACE on skin lesions. The antipsoriatic properties of the ACE cream were evaluated using an imiquimod- (IMQ-) induced psoriasis-like mouse model. In psoriasis-like mouse models, the cumulative score (redness, thickness, and scaling) of the IMQ + ACE cream group was significantly lower than those of the other groups on day 4 (*p* < 0.05). The results of the hematoxylin and eosin staining of skin tissues revealed that the epidermal thickness value of the IMQ + ACE cream group was significantly lower than those of the other experimental groups (*p* < 0.05). The expression level of intracellular adhesion molecule-1 (ICAM-1), which indicates the leukocyte infiltration into the skin and subsequent interactions with keratinocytes, was also lower in the IMQ + ACE cream group than in the IMQ group. These results indicate that ACE cream formulation could be used safely and conveniently for psoriasis treatment.

## 1. Introduction

Psoriasis is an autoimmune disease characterized by itchy, red, and scaly skin patches [1]. There are several main types of psoriasis: plaque, pustular, inverse, napkin, and guttate. The pathogenesis of psoriasis involves the abnormally rapid growth of the skin epidermis. Fast replacement of psoriatic skin cells compared to normal cells may be due to the presence of premature keratinocytes, resulting from inflammatory cascades in the dermis [2]. The transfer of immune cells (i.e., dendritic, macrophage, and T cells) from the dermis to the epidermis and secretion of cytokines (i.e., interleukin- [IL-] 1*β*, IL-6, and IL-22 and tumor necrosis factor- [TNF-] *α*) may stimulate the proliferation of keratinocytes [3]. DNA, which can be released from dying cells, may act as an inflammatory stimulus in psoriatic lesion and could lead to the secretion of cytokines (i.e., IL-1, IL-6, and TNF-*α*) from keratinocytes [3]. The increase of dendritic cells in psoriatic lesions and its involvement in the proliferation of T cells and type 1 helper T cells may be one of mechanisms of psoriasis development [4].

Topical medicines are considered the first choice for mild-to-moderate psoriasis. Corticosteroids, vitamin D analogs, salicylic acid, calcineurin inhibitors, and topical retinoids have been widely used as topical agents [5, 6]. Ultraviolet (UV) light (UVA and UVB) has often been used to treat moderate to severe psoriasis as a phototherapeutic method [5]. Orally administered drugs (e.g., methotrexate, cyclosporine, and acitretin) have been used as a systemic therapeutic approach [5]. TNF-*α* antagonists (e.g., adalimumab, etanercept, and infliximab), monoclonal antibodies (mAb) of the p40 subunit of IL-12 and IL-23 (e.g., ustekinumab), and anti-IL-17 agents (e.g., secukinumab) have been used as targeted immunosuppressive methods [5]. Recently, Janus kinase (JAK) inhibitors (e.g., tofacitinib and baricitinib), phosphodiesterase 4 (PDE4) inhibitors (e.g., apremilast), vitamin A derivatives (e.g., alitretinoin), adenosine A3 receptor antagonists, oxidized phospholipids, fumaric acid derivatives, and sphingosine 1-phosphate receptor-1 (SIP_1_) modulators (e.g., ponesimod) have been developed as emerging therapeutic compounds [5]. Except for synthetic and biological agents, several natural product-based (e.g.*, Aloe vera*,* Baphicacanthus cusia*,* Capsicum frutescens*,* Curcuma longa*,* Hypericum perforatum*,* Indigo naturalis*,* Mahonia aquifolium*,* Strobilanthes formosanus*, and* Persea americana*) formulations have shown antipsoriatic activities [7].

In our previous study [8], the antipsoriatic activities of* Artemisia capillaris* (AC) extract (ACE) were demonstrated in HaCaT cells (a spontaneously transformed aneuploid immortal keratinocyte cell line) and an imiquimod- (IMQ-) induced psoriasis-like mouse model. However, the poor water-solubility of ACE might restrict its suitability for topical application. Although organic solvents (e.g., alcohols) could be used to solubilize the diverse ingredients in ACE, their clinical use may induce toxicity. Therefore, a cream formulation of ACE was prepared for clinical application. Cream formulations have been widely used for topical and transdermal delivery of herbal medicines [9–11]. In this study, the antipsoriatic potential of ACE cream was evaluated in a mouse model by evaluating the severity of psoriasis symptoms and the histological staining patterns.

## 2. Materials and Methods

### 2.1. Materials

6,7-Dimethoxycoumarin (scoparone, 98% purity) was purchased from Sigma-Aldrich Corp., (St. Louis, MO, USA). Chlorogenic acid (≥ 98% purity), 3,5-dicaffeoylquinic acid (≥ 98% purity), and 4,5-dicaffeoylquinic acid (≥ 97% purity) were obtained from ChemFaces (Wuhan, Hubei, China). IMQ cream (Aldara®, 5%) was acquired from 3M Pharmaceuticals (Leicestershire, UK). Tacrolimus (TAC) ointment (Protopic®, 0.1%) was purchased from Astellas Pharma Inc. (Tokyo, Japan). Phosphate-buffered saline (PBS) was obtained from Gibco Life Technologies, Inc. (Grand Island, NY, USA). All solvents were of high-performance liquid chromatography (HPLC) grade and the other chemicals were of analytical grade.

### 2.2. Preparation of ACE

AC was purchased from a local market in Yeongcheon (Gyeongsangbuk-do, Korea) and the ACE was prepared and provided by Radiant. Inc., (Chuncheon, Korea) as reported [8]. AC was identified by Prof. Heejung Yang (Kangwon National University, Chuncheon, Korea). For the extract preparation, fresh AC (20 kg) was added to 70% (v/v) ethanol (EtOH, 200 L) and heated at 65–70°C for 3 h. The resulting extract was filtered through a polypropylene membrane and the organic solvent was removed using a rotary evaporator. The extracted materials were lyophilized and stored for further use.

### 2.3. Preparation and Characterization of ACE Cream Formulation

A cream formulation was developed for the skin delivery of ACE (2%, w/w) and was kindly provided by Hankook Korus Pharm Co., Ltd. (Chuncheon, Korea). The contents of four representative markers of ACE in the cream formulations were quantitatively determined according to a previously reported method [8]. The stock solutions of chlorogenic acid, 3,5-dicaffeoylquinic acid, 4,5-dicaffeoylquinic acid, and 6,7-dimethoxycoumarin were prepared by dissolving each compound in methanol to a concentration of 1 mg/mL. ACE cream (300 mg) was dissolved in methanol (10 mL) and was filtered through a syringe filter (0.45-*μ*m pore size). The sample and standards were simultaneously analyzed using an HPLC system consisting of an autosampler, column oven, pump, and ultraviolet-visible (UV/Vis) detector (all Agilent 1260, Agilent, Santa Clara, CA, USA). The mobile phases A and B were composed of water containing 0.1% (v/v) formic acid and acetonitrile, respectively (B). The flow rate was set at 1.0 mL/min and the injection volume was 10 *μ*L. The gradient program for the HPLC analysis was as follows: (1) 0 min, A:B = 90:10; (2) 12 min, A:B = 88:12; (3) 17 min, A:B = 84:16; (4) 40 min, A:B = 75:25; (5) 50 min, A:B = 62:38; (6) 50.1 min, A:B = 90:10; and (7) 60 min, A:B = 90:10. The HECTOR C18-M column (C18, 4.6 × 250 mm, 5-*μ*m pore size, RStech Co., Ltd., Cheongju, Korea) was used, maintained at a temperature of 35°C in the column oven. The absorption of each sample was detected at 330 nm using a UV/Vis detector.

### 2.4. Establishment of IMQ-Induced Psoriasis-Like Mouse Model

For the animal experiments, female BALB/c mice (8-week-old) were acquired from Orient Bio Inc. (Sungnam, Korea). They were housed in a light-controlled room at a temperature and relative humidity of 22 ± 2°C and 55 ± 5%, respectively, and were provided water and food* ad libitum*. The animal experiments were performed according to the UK Animal (Scientific Procedures) Act 1986 and the National Institute for Health (NIH) Guide for the Care and Use of Laboratory Animals (NIH Publication No. 80-23, revised 1978). The experimental protocols were approved by the Animal Care and Use Committee of Kangwon National University. A previously reported method [8, 12] with slight modifications was used to establish the IMQ-induced psoriasis-like mouse model. The dorsal skin of each mouse was shaved, and IMQ cream was topically applied at a dose of 62.5 mg/day from day 0 to day 4 consecutively to induce psoriasis in the skin.

### 2.5. Evaluation of ACE Cream in Psoriasis-Like Mouse Model

The alleviating effects of ACE cream on psoriasis-like lesion were assessed by comparing it with the pharmacological activities of TAC ointment, ACE suspension, and a blank cream. Each sample was applied to the dorsal skin from day 1 [8]. TAC ointment was topically applied at a dose of 62.5 mg/day from days 1 to 4. ACE was suspended in distilled water (DW) and applied to the dorsal skin at a dose equivalent to 250 mg/kg ACE. ACE cream was applied to the skin at an equivalent dose of 250 mg/kg ACE. The blank cream (without ACE) was also applied to the skin at the same dose. From days 0 to 4, the thickness and erythema values of the dorsal skin were determined using a thickness gauge (digital type, Mitutoyo, Kawasaki, Japan) and Dermacatch® (Colorix, Neuchâtel, Switzerland), respectively. The severity of desquamation was measured by visual inspection. The severity of the psoriasis was presented as the cumulative score by modifying the psoriasis area and severity index (PASI) scores (http://pasi.corti.li, ver. 1.7.1) [8, 12]. Each erythema (redness), induration (thickness), and desquamation (scaling) level was classified into 5 grades (score 0-4) and their cumulative scores were calculated [12].

The skin lesions and spleens were harvested from each mouse on day 4. The spleens were weighed to evaluate the immunological activities of the ACE cream. Dissected skin tissues were washed with PBS at least three times, fixed in 4% (v/v) formaldehyde solution, dehydrated with an alcohol gradient, and then embedded in paraffin for staining. Specimens were cut into 5-*μ*m-thick sections and were stained with hematoxylin and eosin (H&E). The effects of ACE cream on the cell to cell adhesion were evaluated by performing intracellular adhesion molecule-1 (ICAM-1) staining. The immunohistochemistry (IHC) staining was performed using the 3,3′-diaminobenzidine (DAB) development method. The skin tissues were incubated with an ICAM-1 mouse monoclonal antibody (mAb, Santa Cruz Biotechnology, Inc., Dallas, TX, USA) and further processed using the polink-2 plus polymerized horse-radish peroxidase (HRP) DAB detection kit (Golden Bridge International Inc., Mukilteo, WA, USA) according to the manufacturer's protocol. Images were acquired using an inverted microscope (Eclipse TS100, Nikon, Tokyo, Japan).

### 2.6. Statistical Analysis

Each experiment was repeated at least three times, and the experimental data are presented as the mean ± standard deviation (SD). The statistical analysis was performed using Student's* t*-test and analysis of variance (ANOVA).

## 3. Results and Discussion

### 3.1. Preparation and Characterization of ACE Cream

The contents of four markers (chlorogenic acid, 3,5-dicaffeoylquinic acid, 4,5-dicaffeoylquinic acid, and 6,7-dimethoxycoumarin) in ACE were already presented in our previous study [8]. The contents of chlorogenic acid and 3,5-dicaffeoylquinic acid were higher than those of the other two ingredients. In this study, the contents of the four markers in the ACE cream were quantitatively analyzed using an HPLC method (Table 1). Because of the presence of pharmaceutical excipients in the cream formulation, the amounts of the four markers slightly differed from those values previously reported in ACE [8]. Nonetheless, the contents of chlorogenic acid and 3,5-dicaffeoylquinic acid were higher than those of 4,5-dicaffeoylquinic acid and 6,7-dimethoxycoumarin in the ACE cream.

The antipsoriatic effects of ACE were verified in cell culture and animal models in our previous study [8]. However, the poor aqueous solubility of ACE restricted its topical application. ACE contains diverse ingredients with different physicochemical properties (such as solubility); therefore, it was difficult to find suitable solvents for dissolving the whole extract completely. Organic solvents such as alcohols have limitations for clinical use because of their potential toxicities. Therefore, the development of appropriate vehicles for topical administration of ACE is necessary for its clinical application. The ACE cream formulation developed in this study is expected to provide an efficient and safe application of ACE.

### 3.2. Alleviation of Psoriatic Symptoms in Mouse Model

The efficacy of ACE cream in alleviating the symptoms of psoriasis was assessed in an IMQ-induced psoriasis-like mouse model in this study [8]. Topical application of IMQ, which is a ligand of the toll-like receptor (TLR) 7 and 8, induces psoriasis-like dermatitis [12, 13]. This animal model has several different characteristics compared to human psoriasis such as the absence of hypogranulosis and reduced filaggrin [13]. In addition, IMQ-induced psoriasis may be induced by local stimulation and not by systemic immune responses as in human [13]. Nonetheless, it is a very convenient method for establishing a psoriasis-like animal model; thus it has been widely used for assessing the pharmacological activities of potential antipsoriatic agents [13].

To induce psoriasis-like skin lesions, IMQ was topically applied to the mouse skin from day 0 to day 4 in this study. The consecutive daily application of IMQ for that period induced appropriate psoriasis-like skin lesions in the mice in previous reports [8, 14]. In our previous study [8], a TAC formulation was selected as a control treatment for comparing the antipsoriatic effects. Orally administered and topically applied TAC has produced effective outcomes in the treatment of psoriasis [15]. In our previous study [8], ACE was dissolved in 70% (v/v) ethanol before its skin application. For comparing the pharmacological efficacies of the ACE cream formulation in this study, ACE was suspended in DW before application to the psoriatic skin. The blank cream (without ACE loading) was also used as a control treatment.

**Figure 1:**
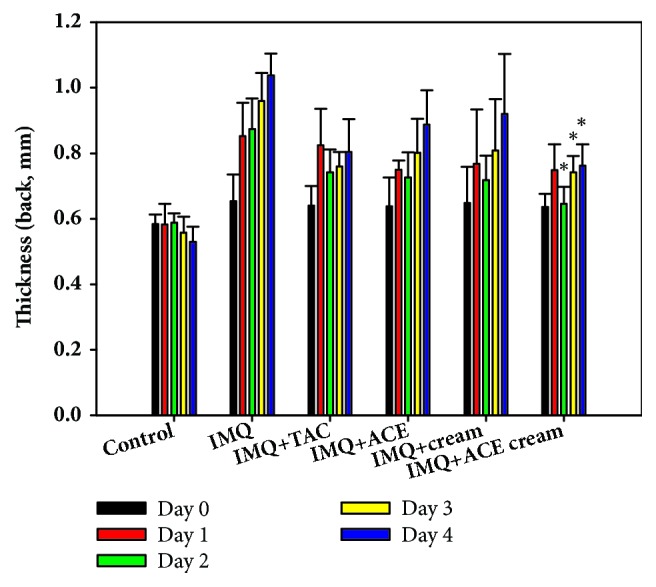
The influences of ACE cream on the thickness of skin in IMQ-induced psoriasis-like mouse models. Thickness (mm) of dorsal skin in each experimental group was measured. Each point indicates the mean ± SD (*n* = 5). ^*∗*^*P* < 0.05, compared with IMQ group.

As shown in Figure 1, the thickness of the dorsal skin lesions (measured by thickness gauge) increased in the IMQ group from day 0 to day 4. The thickness of skin was measured as an independent parameter of inflammation in the skin [12]. The IMQ + ACE cream group exhibited significantly lower thickness values than those of the IMQ group on days 2–4 (*P* < 0.05). The IMQ + ACE and IMQ + cream groups did not show any significant reduction in the thickness of dorsal skin compared with that of the IMQ group. In the IMQ + ACE group, ACE was not completely dissolved in the solvent; thus it did not exert sufficient pharmacological activities. The results of the IMQ + cream group show that the presence of pharmaceutical excipients in the cream formulation did not significantly change the dorsal skin thickness.

**Figure 2:**
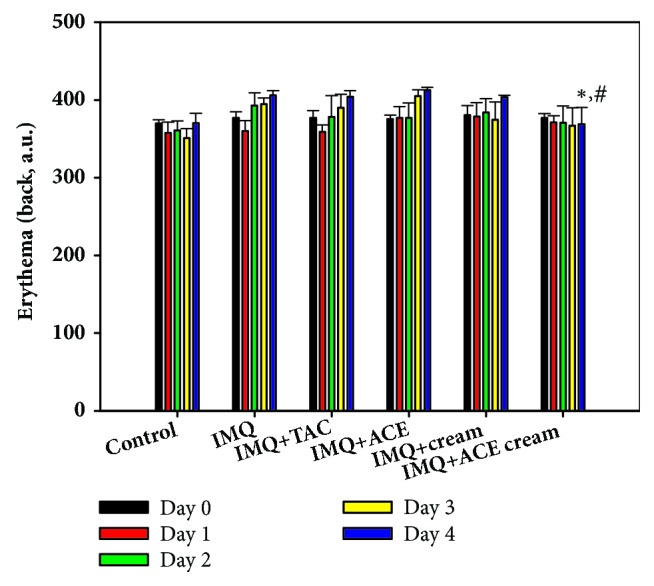
The influences of ACE cream on the erythema level of skin lesions in IMQ-induced psoriasis-like mouse models. Erythema value (a.u.) of dorsal skin in each experimental group was measured. Each point indicates the mean ± SD (*n* = 5). ^*∗*^*P* < 0.05, compared with IMQ group. ^#^*P* < 0.05, compared with IMQ + ACE group.

The erythema levels of the dorsal side were also measured from day 0 to day 4 (Figure 2). In our previous study [8], there was no significant difference between the ACE solution-treated group and other control groups due to the color of the ACE solution. In this study, the erythema level of the IMQ + ACE cream group was significantly lower than those of the IMQ and IMQ + ACE groups on day 4 (*P* < 0.05). The color induced by the ACE content (2%) in the ACE cream formulation appeared to have negligible effects on the erythema levels. The reduction of the erythema level in the IMQ+ACE cream group, compared with IMQ group, indicates the alleviation of psoriatic symptoms of skin lesions.

**Figure 3:**
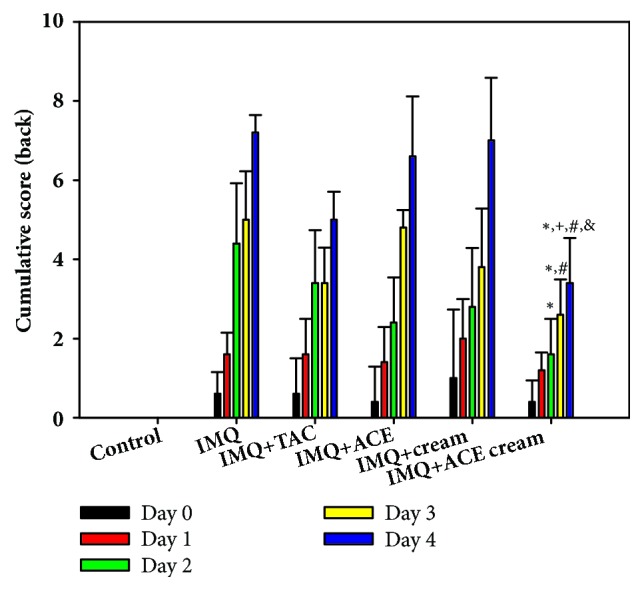
The influences of ACE cream on the cumulative scores in IMQ-induced psoriasis-like mouse models. Cumulative score of dorsal skin in each experimental group was calculated. Each point indicates the mean ± SD (*n* = 5). ^*∗*^*P* < 0.05, compared with IMQ group. ^+^*P* < 0.05, compared with IMQ + TAC group. ^#^*P* < 0.05, compared with IMQ + ACE group. ^&^*P* < 0.05, compared with IMQ + cream group.

The cumulative scores of all experimental groups were calculated by evaluating the erythema (redness), induration (thickness), and desquamation (scaling) (Figure 3) [8, 12]. Each score (from 0 to 4) was determined independently based on PASI scoring concept and the sum of three values was presented (Figure 3). On day 3, the cumulative score of the IMQ + ACE cream group was significantly lower than those of the IMQ and IMQ + ACE groups (*P* < 0.05). Notably, on day 4, the cumulative score of the IMQ + ACE cream group was significantly lower than those of the other groups (*P* < 0.05). These findings indicate that topical application of the developed ACE cream may decrease psoriatic symptoms overall.

**Figure 4:**
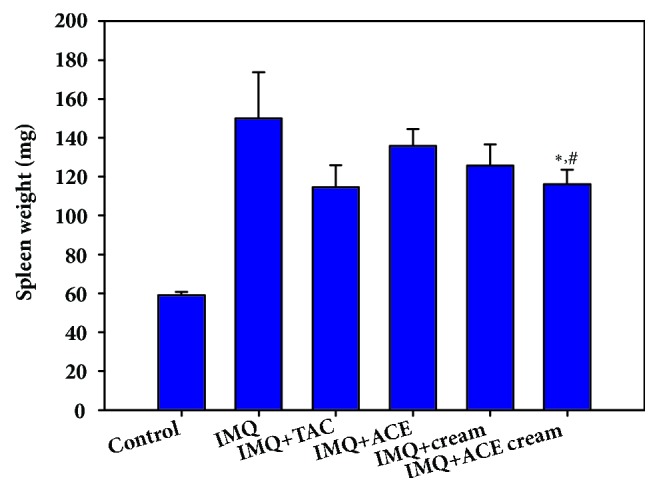
The change of spleen weight after treating ACE cream on IMQ-induced psoriasis-like mouse models. The weight of spleen (mg) in each experimental group was measured. Each point indicates the mean ± SD (*n* = 5). ^*∗*^*P* < 0.05, compared with IMQ group. ^#^*P* < 0.05, compared with IMQ + ACE group.

The weight of the spleen was measured after dissection on day 4, as shown in Figure 4. After multiple applications of IMQ on the dorsal mouse skin for 4 days, the spleen weight of IMQ-treated mice was 2.5-fold higher than that of the untreated control group. The topical application of IMQ has been reported to likely increase the spleen mass and alter its cell composition [16]. The increment in the weight of spleen indicates the increase of cells in the spleen and the elevation of immune reactions in the body [17]. The spleen weight of the IMQ + ACE cream group was significantly lower than those of the IMQ and IMQ + ACE groups (*P* < 0.05). In contrast to the ACE suspension, the developed ACE cream formulation may alleviate the IMQ-induced splenomegaly and change the composition of immune cells in the spleen [18]. Homogeneous suspension of ACE in cream formulation, compared with ACE suspension in DW, and its effective skin application can explain those observed data (Figure 4).

**Figure 5:**
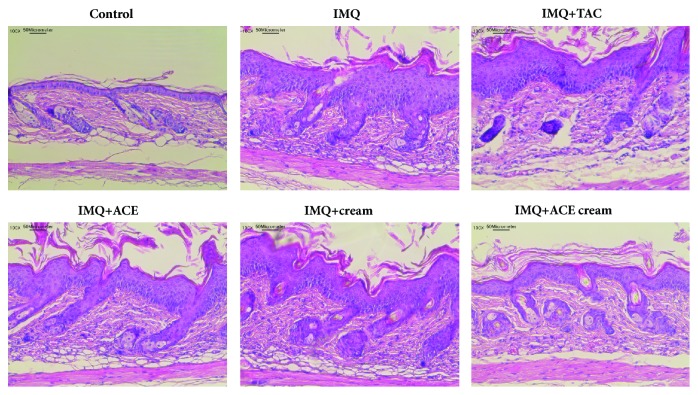
H&E staining of dissected skin tissues. Images of skin tissues stained by H&E are presented. The length of scale bar is presented in the image.

**Figure 6:**
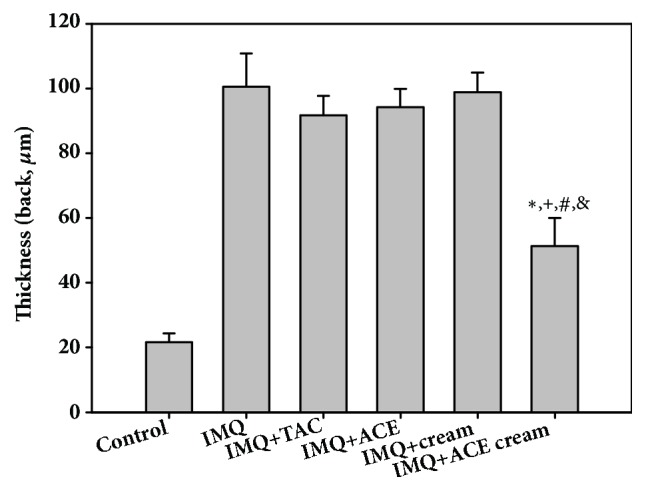
Epidermal thickness (*μ*m) of dissected skin tissues observed in H&E-stained images. Each point indicates the mean ± SD (*n* = 5). ^*∗*^*P* < 0.05, compared with IMQ group. ^+^*P* < 0.05, compared with IMQ + TAC group. ^#^*P* < 0.05, compared with IMQ + ACE group. ^&^*P* < 0.05, compared with IMQ + cream group.

The morphology of the skin lesions and the epidermal thickness were observed using H&E staining (Figures 5 and 6). The epidermal thickness of the IMQ group was 4.7-fold higher than that of the control (no treatment) group. Epidermal hyperplasia and stratum corneum thickening (acanthosis and hyperkeratosis, respectively) were obvious in the IMQ group [19]. The abnormal differentiation of the epidermis and infiltration of leukocytes were also observed in the IMQ group as previously reported [16]. As shown in Figure 6, the epidermal thickness of the IMQ + ACE cream group was significantly lower than those of the IMQ, IMQ + TAC, IMQ + ACE, and IMQ + cream groups (*P* < 0.05). Particularly, the epidermal thickness of the IMQ + ACE cream group was 51% of that of the IMQ group. Although TAC is one of the immunosuppressive agents, its influences on the reduction of epidermal thickness (measured by H&E staining) were not so significant in this tested condition (Figure 6). However, cumulative score (day 4) and spleen weight of IMQ+TAC group were lower than those of IMQ group (Figures 3 and 4). Dosage regimen (i.e., drug dose, dosing interval, and treatment duration) of TAC may determine the therapeutic efficacies for psoriasis-like symptoms [20]. ACE exhibited antiproliferation potential, mainly related to apoptosis, in HaCaT cells in our previous report [8]. It is expected that the inclusion of ACE in the cream may downregulate epidermal proliferation.

**Figure 7:**
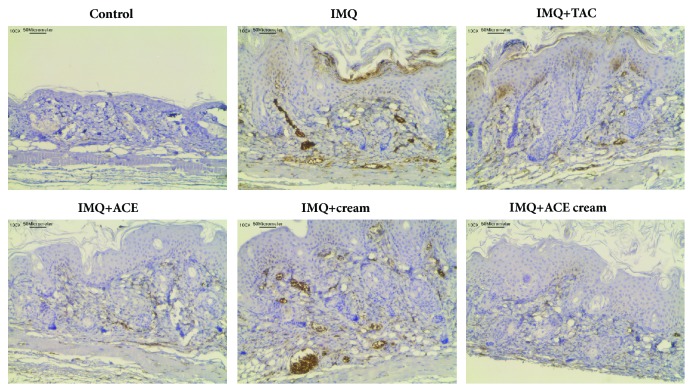
ICAM-1 expression in dissected skin tissues after treating ACE cream in psoriasis-like mouse models. IHC staining results of skin tissues are shown. Brown color (developed by DAB) indicates the ICAM-1 expression in skin tissues. The length of scale bar is presented in the image.

Following the H&E staining of the skin lesions (Figures 5 and 6), the expression level of ICAM-1 was assessed in the skin tissues using IHC staining (Figure 7). The brown color (developed by DAB) indicated that ICAM was expressed in the excised skin tissues. Large portion of brown color was seen in the IMQ group but not in the control (no treatment) group (Figure 7). Infiltration of leukocytes into the skin is considered one of features of psoriasis [21]. Infiltration of leukocytes in the psoriatic skin lesions may be accelerated by the upregulation of ICAM-1 expression. High expression levels of ICAM-1 were frequently observed in the keratinocytes of the psoriatic skin lesions. The ICAM-1 expression level may provide crucial information on the movement of leukocytes and their interactions with keratinocytes [22]. Treatment with TAC or ACE reduced ICAM-1 expression level compared to IMQ treatment alone in this study (Figure 7). There was no significant difference in ICAM-1 expression between the IMQ and IMQ + cream groups. Pharmaceutical excipients included in the blank cream formulation did not significantly affect the ICAM-1 expression level in IMQ-induced psoriasis models. Interestingly, the IMQ + ACE cream group exhibited lower expression levels of ICAM-1 in skin lesions than the IMQ group did. It is expected that the topical application of ACE cream may reduce the inflammatory immune responses in psoriasis.

Generally, the developed ACE cream formulation efficiently alleviated psoriatic symptoms better than the ACE suspension and TAC ointment. The TAC formulation may induce other unwanted effects by depressing the immune systems. The ACE suspension did not show improved antipsoriatic activities compared to IMQ treatment alone due to its incomplete solubilization in DW and subsequent insufficient deposition in the skin lesions. In contrast to the ACE suspension (in water or aqueous buffers) and solution (in organic solvents), the ACE cream formulation can be conveniently and safely used for clinical manifestations with sufficient pharmacological activity.

## 4. Conclusion

The antipsoriatic efficacies of ACE cream were evaluated in an IMQ-induced psoriasis-mimicking mouse model. To overcome the restriction of the low aqueous solubility of ACE, a cream formulation was developed for its topical application. In the psoriasis mouse model, the cumulative score of the IMQ + ACE cream group was significantly lower than those of the other groups on day 4. The weight of the spleens harvested from the IMQ + ACE cream group on day 4 was also lower than those of the IMQ and IMQ + ACE groups. Interestingly, the IMQ + ACE cream group exhibited lower expression level of ICAM-1 in skin lesions than that of the IMQ group. These findings suggest that the ACE cream can be used efficiently and safely for the alleviation of psoriatic symptoms.

## Figures and Tables

**Table 1:** The contents of four representative markers in the ACE cream.

Standards	Content (*μ*g/g)

Chlorogenic acid	374.4 ± 13.1

3,5-Dicaffeoylquinic acid	325.3 ± 30.2

4,5-Dicaffeoylquinic acid	204.7 ± 23.2

6,7-Dimethoxycoumarin	85.5 ± 1.8

The content of ACE in ACE cream is 2% (w/w).

The weight ratio of each marker to cream is presented.

Data are presented as the mean ± standard deviation (SD) (*n *≥ 3).

## Data Availability

Supporting data of this study are included within the article.
